# Lymphocytopenia as a Predictor of Mortality in Patients with ICU-Acquired Pneumonia

**DOI:** 10.3390/jcm8060843

**Published:** 2019-06-13

**Authors:** Adrian Ceccato, Meropi Panagiotarakou, Otavio T. Ranzani, Marta Martin-Fernandez, Raquel Almansa-Mora, Albert Gabarrus, Leticia Bueno, Catia Cilloniz, Adamantia Liapikou, Miquel Ferrer, Jesus F. Bermejo-Martin, Antoni Torres

**Affiliations:** 1 Pneumology Department, Respiratory Institute (ICR), Hospital Clinic of Barcelona - Institut d’Investigacions Biomèdiques August Pi i Sunyer (IDIBAPS) - University of Barcelona, Ciber de Enfermedades Respiratorias (CIBERES), ICREA Academia, 08036 Barcelona, Spain; aceccato@clinic.cat (A.C.); otavioranzani@yahoo.com.br (O.T.R.); gabarrus@clinic.cat (A.G.); bueno@clinic.cat (L.B.); cilloniz@clinic.cat (C.C.); miferrer@clinic.cat (M.F.); 2 Respiratory Department, Sotiria Chest Diseases, 115 27 Athens, Greece; mpanagiotarakou@gmail.com (M.P.); liapikou.adamanthia@gmail.com (A.L.); 3 Respiratory Intensive Care Unit, Pulmonary Division, Heart Institute (InCor), Hospital das Clinicas HCFMUSP, Faculdade de Medicina, Universidade de Sao Paulo, Sao Paulo 01246-903, Brazil; 4 Group for Biomedical Research in Sepsis (Bio∙Sepsis), Grupo de Investigación Biomédica en Sepsis (Bio∙Sepsis), Instituto de Investigación Biomédica de Salamanca (IBSAL), Paseo de San Vicente, 58-182, 37007 Salamanca, Spain; mmartin.iecscyl@saludcastillayleon.es (M.M.-F.); ralmansa@saludcastillayleon.es (R.A.-M.); jfbermejo@saludcastillayleon.es (J.F.B.-M.)

**Keywords:** intensive care unit-acquired pneumonia, mortality, lymphocytes, infection, host response

## Abstract

Background: Intensive care unit-acquired pneumonia (ICU-AP) is a severe complication in patients admitted to the ICU. Lymphocytopenia is a marker of poor prognosis in patients with community-acquired pneumonia, but its impact on ICU-AP prognosis is unknown. We aimed to evaluate whether lymphocytopenia is an independent risk factor for mortality in non-immunocompromised patients with ICU-AP. Methods: Prospective observational cohort study of patients from six ICUs of an 800-bed tertiary teaching hospital (2005 to 2016). Results: Of the 473 patients included, 277 (59%) had ventilator-associated pneumonia (VAP). Receiver operating characteristic (ROC) analysis of the lymphocyte counts at diagnosis showed that 595 cells/mm^3^ was the best cut-off for discriminating two groups of patients at risk: lymphocytopenic group (lymphocyte count <595 cells/mm^3^, 141 patients (30%)) and non-lymphocytopenic group (lymphocyte count ≥595 cells/mm^3^, 332 patients (70%)). Patients with lymphocytopenia presented more comorbidities and a higher sequential organ failure assessment (SOFA) score at the moment of pneumonia diagnosis. Also, 28-day mortality and 90-day mortality were higher in patients with lymphocytopenia (28-day: 38 (27%) versus 59 (18%), 90-day: 74 (53%) versus 111 (34%)). In the multivariable model, <595 cells/mm^3^ resulted to be an independent predictor for 90-day mortality (Hazard Ratio 1.41; 95% Confidence Interval 1.02 to 1.94). Conclusion: Lymphocytopenia is an independent predictor of 90-day mortality in non-immunocompromised patients with ICU-AP.

## 1. Background

Nosocomial pneumonia is one of the most common healthcare-associated infections [1]. It occurs frequently during hospitalization in intensive care units (ICU), causing severe disease that is associated with high morbidity, mortality, and healthcare costs [2,3,4]. 

ICU-acquired pneumonia (ICU-AP) can develop in patients receiving or not mechanical ventilation (ventilator-associated pneumonia (VAP) and hospital-acquired pneumonia (HAP), respectively) and is usually observed in patients with severe underlying conditions [5]. 

Like severe community-acquired pneumonia (CAP) and sepsis, ICU-AP may present marked immunological changes, with lymphocytopenia being one of the most frequently observed [6,7,8,9]. However, the association of lymphocytopenia at the time of diagnosis with mortality has not been assessed to date. 

A previous study by our group showed that patients with CAP and lymphocyte counts <724 lymphocytes/mm^3^ had a particular immunological phenotype which affected a third of patients and was associated with a twofold increase in 30-day mortality risk [10]. Similar results have been observed in patients with sepsis, in whom decreased lymphocyte counts in the blood were thought to be secondary to the recruitment of these cells to or their escape from sites of inflammation/infection and/or to apoptosis, which depletes all types of lymphocyte [11,12]. 

This study aimed to determine whether a low lymphocyte count at the time of ICU-AP diagnosis is a predictor of lower survival. We evaluated the association between lymphocyte counts in patients with ICU-AP and 90-day mortality risk. We also determined the cut-off values for lymphocyte counts that best predicted 90-day mortality.

## 2. Materials and Methods

### 2.1. Design and Patients

This prospective cohort study evaluated patients diagnosed with ICU-AP in six ICUs of an 800-bed tertiary teaching hospital (Hospital Clinic, Barcelona, Spain), admitted from 2005 to 2016. The six ICUs comprised a broad range of cases, including medical and surgical patients. The investigators made daily rounds in the ICUs, and patients were included in the study if they were aged ≥18 years and presented clinically suspected pneumonia 48 h after ICU admission. Patients with severe immunosuppression were excluded, such as those receiving solid organ or hematopoietic transplantation or chemotherapy, those with drug-induced immunosuppression, or those with HIV infections. Only the first episode of pneumonia in each patient was assessed. 

The study was approved by the Institution’s Internal Review Board (Comite Etic d’Investigacio Clinica, registry number 2009/5427), and written informed consent was obtained from the patients or their relatives.

### 2.2. Definition of Pneumonia

Pneumonia was clinically diagnosed in patients who had new or progressive pulmonary infiltrates on their chest radiographs due to an infectious agent and who had at least two of the following symptoms or findings: fever (>38 °C) or hypothermia (<36 °C), leukocytosis (>12,000 cells/mm^3^) or leukopenia (<4000 cells/mm^3^), presence of purulent tracheal secretions, and a decline in oxygenation [2,13,14]. ICU-AP was defined as pneumonia developed after more than 48 h of ICU admission. VAP was identified in patients who were treated with invasive mechanical ventilation for ≥48 h and had met the above-mentioned criteria; HAP was identified in patients who developed pneumonia after 48 h of ICU-admission, not receiving invasive mechanical ventilation.

### 2.3. Data Collection

All relevant data were collected upon ICU admission and at the onset of pneumonia from medical records and bedside flow charts, including clinical, laboratory, radiological, and microbiological information. Multidrug-resistant pathogens were defined on the basis of consensus [15]. Patient follow-up was extended to death, hospital discharge, or up to 90 days after the diagnosis of pneumonia. Severity was assessed using the sequential organ failure assessment (SOFA) score [16] during ICU admission and at ICU-AP diagnosis. 

### 2.4. Antimicrobial Treatment

Initial empiric antibiotic therapy was administered at the attending physician’s discretion. The local policy and practice were based mainly on a local adaptation of the 2005 ATS/IDSA guidelines [14]. It was also recommended that physicians should base their empiric choices on the local prevalence of multidrug-resistant (MDR) pathogens. The local policy recommended revising the antibiotic regimen on the basis of culture results. We considered that patients who received empiric antibiotic treatment in accordance with the 2005 ATS/IDSA guidelines had been administered appropriate antibiotic treatment [14]. 

### 2.5. Leukocyte and Lymphocyte Quantification

Leukocyte and lymphocyte counts were performed with blood collected in EDTA tubes using the automatic analyzers available and standard operating procedures approved for clinical use.

### 2.6. Outcome Measures

The primary outcome measure was all-cause mortality on day 90 after the onset of ICU-AP. The association of lymphocytopenia with treatment failure was also assessed. Initial response to treatment was evaluated after 72 h of antimicrobial treatment, as previously described [17].

### 2.7. Statistical Analysis

Categorical variables are presented as numbers and percentages of patients, while continuous variables (non-normally distributed data) are presented as medians and interquartile ranges (IQR). Categorical variables were compared with the chi-square test or Fisher’s exact test, while continuous variables in the two groups were assessed using the non-parametric Mann–Whitney U test. A receiver operating characteristic (ROC) curve was constructed to determine the best cut-off point for lymphocyte counts for predicting 90-day mortality. Youden’s index [18] was defined for all the points along the ROC curve, and the maximum value of the index was used as a criterion for selecting the optimum cut-off point. Time to 90-day mortality was analyzed by Kaplan–Meier survival curves and compared using the Gehan–Breslow–Wilcoxon test. Cox proportional hazards regression analyses [19] were performed to determine the effect of risk factors on 90-day mortality. First, each risk factor was tested individually. Finally, factors showing an association in the univariate analyses (*p* < 0.10) were incorporated into the multivariable Cox regression model to predict 90-day mortality. The final selection of the variables was performed using the backward stepwise selection method (likelihood ratio) (*p*_in_ < 0.05, *p*_out_ > 0.10). Lymphocyte counts were corrected using a logarithmic transformation in order to obtain a normal distribution. Hazard ratios (HRs) and their 95% confidence intervals (CIs) were calculated. Proportional hazards assumptions were tested with log-minus-log plots. To investigate the lack of fit of our final model, we evaluated the deviance residuals. The ability of the cut-off value for lymphocyte counts to predict 90-day mortality was further evaluated by using Cox regression analysis. The internal validity of the prediction models was assessed using ordinary non-parametric bootstrapping with 1000 bootstrap samples and bias-corrected, accelerated 95% CIs [20]. Additionally, in order to incorporate nonlinear effects into the Cox regression model, we expressed the hazard as an additive Cox model [21,22]. We used restricted cubic regression splines, and curves were constructed according to degrees of freedom (DF), chosen by Akaike information criteria (df = 4, lowest value). The level of significance was set at 0.05 (two-tailed). All analyses were performed with the IBM SPSS Statistics 25.0 software (Armonk, NY, USA) and R 3.5.3 software (Vienna, Austria).

## 3. Results

### 3.1. Participants

**Figure 1:**
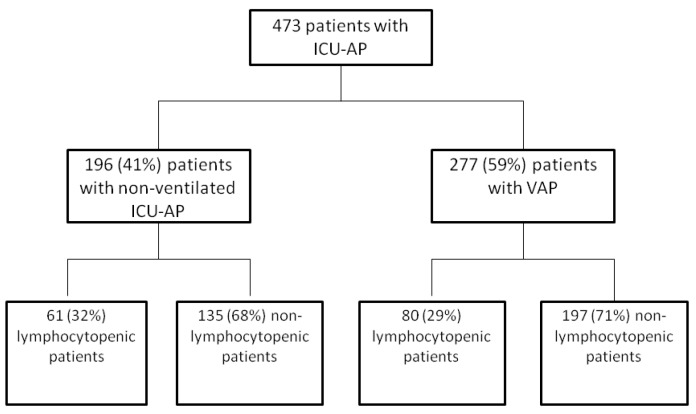
Flowchart. ICU-AP: intensive care unit-acquired pneumonia, VAP: ventilator-associated pneumonia.

Of the 473 patients with ICU-AP included during the study period, 196 (41%) had HAP (Figure 1), and 277 (59%) had VAP. 

### 3.2. Patients Clinical Characteristics

The clinical characteristics of the patients with ICU-AP are presented in Table 1, stratified by the presence of lymphocytopenia (lymphocyte counts <595 cells/mm^3^). On average, patients with lymphocytopenia had more comorbidities such as chronic renal failure, respiratory disease, and chronic liver disease. They were also more likely to be admitted to ICU for hypercapnic respiratory failure or after surgery and presented more severe disease at the onset of pneumonia with higher SOFA score and multilobar involvement. The two groups showed similar rates of treatment failure at 72 h.

### 3.3. Lymphocyte Counts

The median lymphocyte count for the 473 patients with ICU-AP was 821 (528; 1243) cells/mm^3^. Following Youden’s index, we selected 595 cells/mm^3^ as the optimal cut-off point for lymphocyte counts in relation to 90-day mortality (40% sensitivity, 76% specificity, 52% positive predictive value, 67% negative predictive value, 1.69 positive likelihood ratio, and 0.79 negative likelihood ratio) (Figure S1 and Table S1). The patients were divided into two groups: lymphocytopenic group (lymphocyte counts <595 cells/mm^3^, 141 patients (30%) and non-lymphocytopenic group (lymphocyte counts ≥595 cells/mm^3^, 332 patients (70%).

### 3.4. Microbiologic Results of Patients with ICU-AP, According to the Presence of Lymphocytopenia

Microbiological diagnosis was possible in 306 patients (65%), with no differences between patients with or without lymphocytopenia (97 patients (69%) versus 209 patients (63%), respectively; *p* = 0.22). The most common microorganism isolated was *Pseudomonas aeruginosa,* identified in 99 patients (32%) and more frequent in patients with lymphocytopenia than in patients without it (41 (42%) versus 58 (28); *p* = 0.012) (Table 2). The proportion of appropriate empirical treatment did not differ between groups (108 patients (80%) in the lymphocytopenic group versus 255 patients (80%) in the non-lymphocytopenic group; *p* > 0.99)

### 3.5. Outcomes

**Figure 2:**
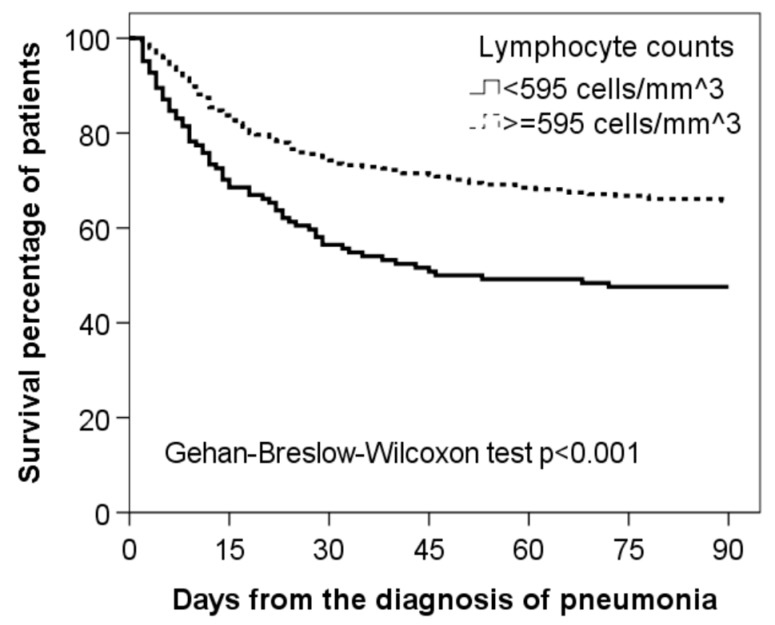
Kaplan–Meier survival curves for 90-day mortality in ICU-AP patients in relation to their lymphocyte counts at diagnosis.

Patients with lymphocytopenia presented higher mortality rates at 28- and 90-days after initial diagnosis (Table 1). Kaplan–Meier curves for 90-day survival according to the presence or absence of lymphocytopenia are shown in Figure 2.

### 3.6. Analysis of 90-Day Mortality Risk

Significant results from the univariate and multivariable analyses are presented in Table 3. Multivariable analysis showed that lymphocyte counts <595 cells/mm^3^ were an independent risk factor for 90-day survival (HR 1.41, 95% CI, 1.02 to 1.94). Internal validation of the final model showed that all variables remained significant after bootstrapping, with small 95% CIs for the original coefficients (Table S2). Other factors associated with 90-day survival in the multivariable analyses were chronic obstructive pulmonary disease (COPD), chronic liver disease, and use of corticosteroids prior to the diagnosis of ICU-AP.

**Figure 3:**
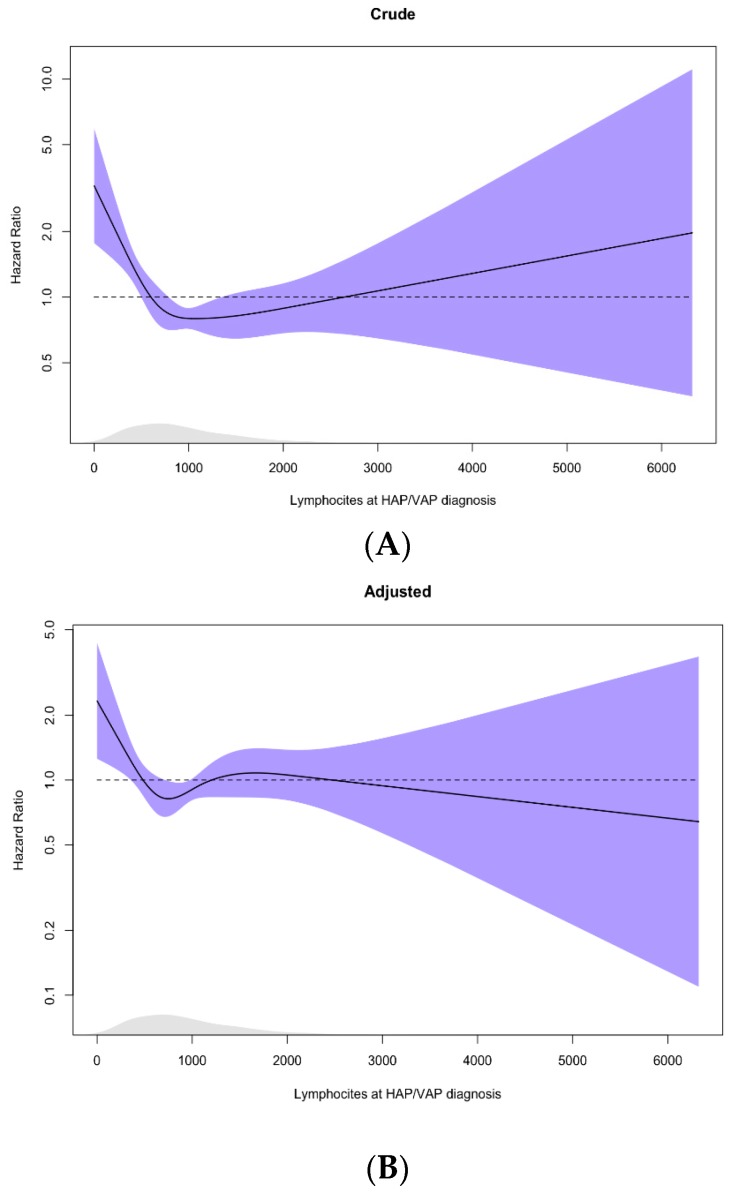
(**A**) Crude and (**B**) adjusted effect of lymphocytes on 90-day mortality. The curves were estimated in Cox proportional hazards models using restricted cubic splines with four degrees of freedom (df); 95% CI in shaded area, Rug density of lymphocytes distribution at the bottom. Adjusted for age, liver, cardiovascular and respiratory chronic diseases, systemic steroids at ICU-AP diagnosis, and SOFA at ICU-AP diagnosis; *p* for nonlinearity, *p*-value crude: < 0.001, *p*-value adjusted: 0.0315.

Figure 3 provides estimates of the exposure–response function of lymphocyte count on 90-day survival, using restricted cubic regression splines. We observed a significant non-linear effect of lymphocyte counts on 90-day survival in crude and adjusted analyses. The increase in the risk of death due to low lymphocyte count was similar to the cut-off value retrieved from the ROC analysis. 

## 4. Discussion

In this analysis of a well-characterized cohort of patients with ICU-AP, we found lymphocyte count to be a predictor of 90-day survival with a non-linear risk. Our results showed that a lymphocyte count below 595 cells/mm^3^ at diagnosis was a robust, independent risk factor for 90-day mortality in these patients. 

ICU-AP is a severe complication that may lead to death [23,24,25,26]. Several risk factors have been described for developing this hospital-acquired form of pneumonia. Although the incidences of VAP and HAP are declining [27,28,29,30], they remain important and contribute to the intensive use of antibiotics and healthcare resources as well as to high mortality rates [30,31]. Despite the differences between the epidemiology of VAP and HAP, both types of pneumonia can be considered as ICU-AP [5,32].

Identifying risk factors for mortality could help to improve patient care and personalize treatments [33]. Pneumonia frequently leads to sepsis and induces significant local and systemic inflammation. Hyper- and hypo-inflammatory responses are observed in patients with sepsis, the hypo-inflammatory response presenting with lymphocytopenia or T-cell exhaustion [6]. The lymphocytopenia observed is not classical immunosuppression; however, differences in immune response with respect to patients with sepsis who died in ICU have been observed [34]. Identifying different endotypes in patients with sepsis is a major challenge at present [35,36,37]. A previous study by our group showed that patients with CAP had a higher risk of mortality if they presented lymphocytopenia. In CAP, the addition of lymphocytopenia to the CURB-65 score increases the latter’s accuracy for predicting mortality. The optimal cut-off point for predicting 30-day mortality was identified as 724 lymphocytes/mm^3^ in patients with CAP [10]. In the present study, the optimal cut-off point for predicting 90-day mortality was lower for ICU-AP than for CAP.

A recent study in critically ill patients revealed that patients with CAP and HAP showed similar inflammatory responses and gene expression profiles [38]. However, patients with HAP exhibited lower expression levels of the genes involved in the type I interferon signaling pathway and increased expression of genes associated with cell junctions and cell movement.

The association between low lymphocyte counts and mortality in ICU-AP might lead to the identification of new therapeutic agents such as drugs that prevent apoptosis, promote cell survival, or activate T cells (i.e., interleukin-7 or anti-programmed cell death protein 1 antibodies). These drugs have been shown to reverse the immunosuppression caused by sepsis in animal models [39,40], and an anti-programmed cell death protein 1 antibody was well tolerated in a phase 1 study [41]. Unfortunately, it is not yet clear whether the presence of low lymphocyte counts has any real impact on patient survival or whether it is merely a biomarker of disease severity. Nonetheless, using lymphocyte counts to identify patients at a higher risk of mortality could help to personalize treatments and to stratify patients by severity in clinical studies.

Other independent predictors of 90-day mortality were COPD, liver disease, and use of corticosteroids at the time of ICU-AP diagnosis. These findings are consistent with previous publications by our group [42,43,44], and, in addition, lymphocytopenia may help to stratify the mortality risk of populations enrolled in randomized controlled trials designed to explore potential differences in treatment effects.

Our study has some limitations. First, the study was carried out at a single center in Spain. However, it involved six different ICUs from the hospital. Second, only ICU patients were included; thus, the profiles of patients with HAP managed outside the ICU may be different. The major strength of our study is the large and well-characterized cohort studied.

## 5. Conclusions

In conclusion, a low lymphocyte count at the moment of diagnosis of ICU-AP increases the risk to die: presenting with less than 595 cell/mm^3^ is a predictor of long-term mortality in patients with this disease. Lymphocyte counting is an inexpensive tool that is widely available in hospitals in both developing and developed countries and could help to personalize treatment in patients with ICU-AP.

## Figures and Tables

**Table 1:** Patients’ clinical characteristics and outcomes.

Variable	Lymphocytopenic Patients *n* = 141	Non-Lymphocytopenic Patients *n* = 332	*p*-Value
Age, years	68 (58; 76)	66 (54; 74)	0.13
Sex, male/female, n	99/42	226/109	0.65
Previous corticosteroid use	26 (20)	30 (10)	0.003
SAPS score at ICU admission	40 (29; 50)	38 (29; 48)	0.49
SOFA score at ICU admission	7 (5; 10)	7 (5; 10)	0.88
Lymphocyte counts at ICU admission, cells/mm^3^	533 (352; 844)	1005 (659; 1382)	<0.001
Co-morbidities			
Diabetes mellitus	40 (28)	72 (22)	0.12
Chronic renal failure	19 (13)	26 (8)	0.056
Solid cancer	27 (19)	56 (17)	0.55
Chronic heart disorders	50 (35)	111 (33)	0.67
Chronic lung disease	56 (40)	98 (30)	0.030
COPD	38 (27)	64 (19)	0.063
Chronic liver disease	39 (28)	51 (15)	0.002
Main causes of ICU admission:			
Post-operative	20 (14)	78 (23)	0.022
Decreased consciousness	17 (12)	40 (12)	>0.99
Hypoxemic respiratory failure	29 (21)	53 (16)	0.23
VAP/HAP	80 (57)/ 61 (31)	197 (59)/ 135 (69)	0.60
Severity of pneumonia assessment			
SOFA score at day 1	8 (6; 10)	7 (4; 9)	0.003
Multi-lobar involvement	77 (55)	143 (43)	0.021
ARDS criteria	21 (15)	41 (12)	0.47
Shock at onset of pneumonia	69 (49)	135 (41)	0.050
Laboratory variables at onset of pneumonia			
Serum creatinine, mg/dL	1 (0.6; 1.7)	0.9 (0.7; 1.4)	0.19
White blood cell count, L^−9^	10,500 (7000; 14,200)	13,560 (9555; 18,250)	<0.001
Lymphocyte counts, cells/mm^3^	396 (268; 589)	1045 (793; 1431)	<0.001
Outcomes			
Treatment failure at 72 h	85 (60)	170 (51)	0.070
28-day mortality	38 (27)	59 (18)	0.024
90-day mortality	74 (53)	111 (34)	<0.001

Abbreviations: ARDS, acute respiratory distress syndrome; VAP, ventilator-associated pneumonia; COPD, chronic obstructive pulmonary disease; CPIS, clinical pulmonary infection score (only for patients with VAP); ICU, intensive care unit; HAP, hospital-acquired pneumonia; PaO_2_/FiO_2_, ratio of partial pressure arterial oxygen to fraction of inspired oxygen; SAPS, simplified acute physiology score; SOFA, sequential organ failure assessment. Data are presented as number of patients (%) or median (1st quartile; 3rd quartile); *p*-values calculated by Mann–Whitney U test, chi-square test, or Fisher’s exact test. Percentages calculated on non-missing data.

**Table 2:** Microbial aetiology.

	Lymphocytopenic Patients *n* = 141	Non-Lymphocytopenic Patients *n* = 332	*p*-Value
Microbiological diagnosis	97 (69)	209 (63)	0.22
*Staphylococcus aureus*	25 (26)	56 (27)	0.851
*Streptococcus pneumoniae*	6 (4)	11 (6)	0.499
*Enterobacteriaceae*	20 (21)	71 (34)	0.017
*Pseudomonas aeruginosa*	41 (43)	58 (28)	0.012
MDR pathogens, *n* (%)	38 (39)	67 (32)	0.222
P. aeruginosa *	15 (9)	21 (36)	0.440
Methicillin-resistant S. aureus (MRSA) *	5 (20)	22 (39)	0.089
Acinetobacter baumannii *	1 (50)	0	-
MDR Enterobacteriaceae *	8 (40)	14 (20)	0.061

Abbreviations: MDR, multidrug resistant. Data are presented as number of patients (%); *p*-values calculated by chi-square test or Fisher’s exact test. Percentages calculated on non-missing data. * Percentage calculated for the total of each pathogen.

**Table 3:** Cox regression analyses to predict 90-day mortality in patients with ICU-AP.

	Univariate ^a^			Multivariable ^b^		
	HR	95% CI	*p*-Value	HR	95% CI	*p*-Value
Age (+1 year)	1.02	1.01–1.04	<0.001	1.03	1.01–1.04	<0.001
Liver disease	2.03	1.47–2.81	<0.001	1.78	1.24–2.54	0.002
Chronic pulmonary disease	1.73	1.29–2.32	<0.001	1.60	1.16–2.19	0.004
Corticosteroids before admission	1.85	1.26–2.72	0.002	-	-	-
Corticosteroids at diagnosis	1.40	1.05–1.87	0.024	1.43	1.04–1.95	0.026
Previous surgery	0.66	0.49–0.89	0.006	-	-	-
SOFA at diagnosis (+1 point)	1.12	1.07–1.17	<0.001	1.13	1.08–1.18	<0.001
Non-ventilated hospital-acquired pneumonia	1.28	0.96–1.71	0.098	-	-	-
Appropriate antibiotic treatment	0.61	0.44–0.84	0.003	-	-	-
Lymphocytes (<595 cells/mm^3^) ^b^	1.83	1.36–2.46	<0.001	1.41	1.02–1.94	0.038

Abbreviations: CI, confidence interval; HR, hazard ratio. Data are shown as estimated HRs (95% CIs) of the explanatory variables in the 90-day mortality group. The HR is defined as the ratio of the hazard rates corresponding to the conditions described by two levels of an explanatory variable (the hazard rate is the risk of death (i.e., the probability of death), given that the patient has survived up to a specific time). The *p*-value is based on the null hypothesis that all HRs relating to an explanatory variable equal unity (no effect). ^a^ The variables analyzed in the univariate analysis were: age, gender, diabetes mellitus, chronic renal disease, neoplasia, chronic cardiovascular disease, chronic liver disease, chronic pulmonary disease, corticoids before admission, corticoids at diagnosis, previous surgery, SOFA score, appropriate antibiotic treatment, and ventilator-associated pneumonia. ^b^ Optimal cut-off value to predict 90-day mortality using receiver operating characteristic (ROC) curves.

## Supplementary Materials

The following are available online at https://www.mdpi.com/2077-0383/8/6/843/s1, Figure S1: Area under the receiver operating characteristic curves (AUCs) for prediction of 90-day mortality A: Lymphocytes count and B: SOFA score; Table S1: Predictive performance of lymphocyte counts for 90-day mortality in patients with ICU-AP; Table S2. Internal validation of the prediction model for 90-day mortality adjusted for lymphocytes (<595 cells/mm^3^) by the nonparametric bootstrap technique.

## Declarations

The datasets used and/or analyzed during the current study are available from the corresponding author on reasonable request.

## Abbreviations

CAP	community-acquired pneumonia
95%CIs	95% confidence intervals
DF	degrees of freedom
HAP	hospital-acquired pneumonia
HRs	hazard ratios
ICU	Intensive care units
ICU-AP	ICU-acquired pneumonia
IQR	interquartile range
SOFA	sequential organ failure assessment
VAP	ventilator-associated pneumonia
