# Supplementary material for: Lymphocytopenia as a Predictor of Mortality in Patients with ICU-Acquired Pneumonia
Source: J Clin Med. 2019 Jun 13;8(6):843. doi: 10.3390/jcm8060843 (PMC6617552; doi:10.3390/jcm8060843)
Supplement: Supplementary file 1 [file jcm-08-00843-s001.pdf]

Table S1. Predictive performance of lymphocyte counts for 90-day mortality in patients with ICU-AP

	Value	95% Confidence Interval	
		Inferior	Superior
Sensitivity (%)	40	32	47
Specificity (%)	76	71	82
Positive predictive value (%)	52	43	60
Negative predictive value (%)	67	61	72
Youden Index	0.16	0.08	0.25
Positive likelihood ratio	1.69	1.28	2.22
Negative likelihood ratio	0.79	0.69	0.90

Table S2. Internal validation of the prediction model for 90-day mortality adjusted for the lymphocytes (<595 cells/mm^3) by the nonparametric bootstrap technique

Variable	Original	Bias	SE	P-value	95% BCa CI
Lymphocytes (<595 cells/mm^3)	0.340	0.006	0.167	0.043	-0.012 to 0.706
Age (+1 year)	0.028	0.000	0.007	0.001	0.014 to 0.044
Liver disease	0.574	-0.001	0.185	0.002	0.184 to 0.925
Chronic pulmonary disease	0.467	0.001	0.163	0.002	0.132 to 0.795
Corticosteroids at diagnosis	0.356	-0.003	0.168	0.031	0.034 to 0.689
SOFA at diagnosis (+1 point)	0.119	0.002	0.023	0.001	0.071 to 0.169

Abbreviations: BCa, adjusted bootstrap; CI, confidence interval; SE, standard error.

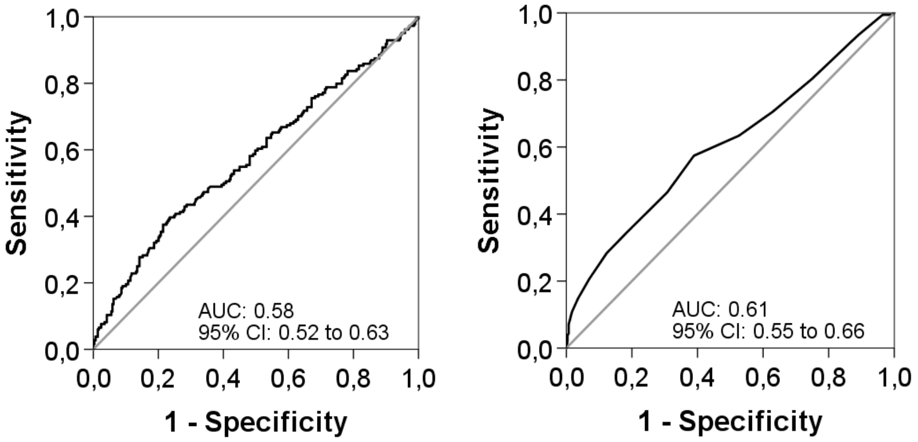

Figure S1. Area under the receiver operating characteristic curves (AUCs) for prediction of 90-day mortality A: Lymphocytes count and B: SOFA score
